# Lower Urinary Tract Symptoms (LUTS) as a Risk Factor for Depressive Symptoms in Elderly Men: Results from a Large Prospective Study in Southern Chinese Men

**DOI:** 10.1371/journal.pone.0076017

**Published:** 2013-09-30

**Authors:** Roger Y. Chung, Jason C. S. Leung, Dicken C. C. Chan, Jean Woo, Carmen K. M. Wong, Samuel Y. S. Wong

**Affiliations:** 1 School of Public Health and Primary Care, The Chinese University of Hong Kong, Hong Kong SAR, China; 2 Jockey Club Centre for Osteoporosis Care and Control, The Chinese University of Hong Kong, Hong Kong SAR, China; 3 Department of Medicine and Therapeutics, The Chinese University of Hong Kong, Hong Kong SAR, China; National Taiwan University, Taiwan

## Abstract

A cross-sectional relationship between lower urinary tract symptoms (LUTS) and depressive symptoms was previously reported among Southern Chinese men; however, the temporal relationship was unclear. Our objective is to evaluate the temporal relationship between moderate to severe lower urinary tract symptoms and clinically significant depressive symptoms in elderly Chinese men aged 65 in a prospective manner. In a prospective cohort of 2,000 Chinese men aged 65 to 92 years in Hong Kong, we studied the association of having moderate to severe LUTS at baseline and having clinically relevant depressive symptoms at year 2 follow-up. After excluding men with prostate or bladder cancer or surgery (n = 20) and lost to follow-up (n = 254), data on 1,726 subjects were analyzed. LUTS were measured by the International Prostate Symptom score; and clinically relevant depressive symptoms were measured by the Geriatric Depression Scale. The multiple logistic regressions showed that the presence of moderate-to-severe LUTS at baseline were significantly associated with increased risk for being depressed at two-year follow-up, with adjustments for demographic, lifestyle, medical factors, weight status and stressful life events (OR = 2.97; CI: 1.70–5.20). Association remained significant with additional adjustments for baseline GDS score (OR = 1.88; CI: 1.03–3.41). LUTS are important risk factors in predicting the presence of clinically relevant depressive symptoms. In elderly men, increased awareness and possible screening are needed to detect the increased risk of clinically relevant depressive symptoms.

## Introduction

It has been established that depressive symptoms or depression are related to a number of chronic medical conditions including cardiovascular diseases [1], chronic obstructive pulmonary diseases (COPD) [2], congestive heart diseases [3] and other debilitating conditions. Results from these previous studies showed that the presence of chronic medical conditions was associated with increased risk of having clinically relevant depressive symptoms or depression. In terms of public health impact, it was shown that the presence of depressive symptoms among people with chronic conditions are associated with increased disability, morbidity and mortality [3]–[5].

Lower urinary tract symptoms (LUTS) are common chronic conditions in elderly men. Previous large epidemiological studies showed that the prevalence of LUTS ranges from 40% to 80% in men aged over 50, and increases with increasing age [6], [7]. Studies have also shown a decreased quality of life in men, poorer role functioning, and poorer emotional outcomes associated with LUTS [8]–[14]. Although the prevalence of LUTS is high and the impact is significant, few prospective studies have been carried out to evaluate the association between LUTS and depressive symptoms which are important to delineate the temporal and causal relationships between LUTS and clinically relevant depressive symptoms.

We previously reported the relationship between LUTS and clinically relevant depressive symptoms using data from a cross-sectional study [15]. However, the temporality of the relationship has not been established. The current study was conducted to delineate the temporal relationship between having LUTS and depressive symptoms. Moreover, the relationship between LUTS and clinically relevant depressive symptoms was studied among other demographic, lifestyle and medical factors.

## Materials and Methods

### Ethics Statement

This study was approved by the Clinical Research Ethics Committee of the Chinese University of Hong Kong, and all the study participants gave written informed consent.

### Study Subjects

The methodology of this study was described previously [16]. In brief, 2,000 Chinese men who were 65 years and older were recruited for this study. To be eligible, subjects had to be able to walk independently. Stratified sampling was adopted in order to have around 33% of subjects in each of the following age groups: 65–69, 70–74, ≥75 years. Recruitment notices were placed in housing estates and community centers for the elderly in Hong Kong. Subjects were invited to the research center for interviews and physical examination. A comprehensive standardized questionnaire was used to collect the information needed at baseline. After two years (i.e., at Year 2), the subjects were then invited again for a second visit to answer repeated questions for measuring clinically relevant depressive symptoms. Informed consent was obtained from all subjects.

For this analysis, men with a history of bladder or prostate cancer or surgery for bladder cancer were excluded.

### Questionnaire

Subjects were interviewed from August 2001 to February 2003 using a standardized, structured questionnaire which covered the following aspects. They were then followed up after two years with some of the questions asked at baseline repeated. Details of the questionnaire items are listed below.

#### Demographic characteristics

Information on age, residential address, place of origin, education levels and occupation was obtained.

#### Medical conditions and medication use

Information on subjects’ medical history was obtained based on self-reports of medical conditions. Subjects were asked to bring in all medications for identification.

#### Lower Urinary Tract Symptoms (LUTS)

Each subject was asked to grade the severity of LUTS using the Chinese version of the International Prostatic Symptoms Score (IPSS) [17] for LUTS. Seven questions of the IPSS assess the severity of the symptoms. These symptoms include nocturia, frequency, urgency, intermittency, weak stream, incomplete emptying and straining. The IPSS is the sum of all seven scores with a range of 0 to 35. A score of 1 or more defined a respondent to have that particular symptom.

Using standard cut-points for symptom severity, men were defined as having severe LUTS if they scored 20 or more on the IPSS. They were defined as having moderate LUTS if they scored from 8 to 19. Mild LUTS were defined as having a score of 7 or less.

#### Clinically relevant depressive symptoms

Questions on depression were asked both at baseline and at Year 2. Depression was diagnosed by a trained interviewer during face-to-face interviews, using a validated 15-item Chinese version of the Geriatric Depression Scale (GDS), with depression being defined as scoring 8 or above [18]. The use of the short form was found to be a useful measure and screening tool for geriatric depression due to the elderly tendency to fatigue with long questionnaire. The GDS short form was found to be a highly reliable (reliability coefficient of 0.90) and valid screening device (sensitivity of 96.3% and specificity of 87.5%) for assessing geriatric depression in Hong Kong [18].

#### Cigarette smoking and alcohol consumption

Cigarette smoking and alcohol consumption were based on self-report using validated methods [19]. Information on the duration and dosage of past and current use of cigarettes, cigars and pipes were obtained. Smoking status was defined as former smoker (at least 100 cigarettes smoked in a lifetime), current smoker, and those who never smoke. For current smokers, the number of cigarettes smoked per day over the previous 12 months was categorized as <20 or ≥20.

For alcohol consumption, subjects were asked to report their daily frequency of intake of alcohol and other beverages in portion sizes. They were also asked to report on how many days of the week they consumed alcohol. Drinking status was categorized as never, former or current drinker. Current drinkers were defined as those who drank at least 12 drinks of beer, wine (including Chinese wine), or liquor over the previous 12 months. For estimation of total alcohol intake in grams per day, servings of specified types of alcoholic beverages were multiplied by the number of grams per serving. A standard drink was one that contained 10 grams of alcohol.

#### Physical activity

Physical activity was measured by the validated Physical Activity Scale for the Elderly Questionnaire (PASE) [20]. It measured the level of physical activity in individuals aged 65 years and older. The instrument is a self-report interview-based measure designed to capture and assess occupational, household, and leisure activities typically performed by older adults. PASE was adapted for the Hong Kong Chinese population by adding activity items that were popular among the local elderly.

### Statistical Analysis

Crude relative risk and corresponding 95% confidence intervals were calculated for examination of associations between moderate to severe LUTS at baseline and clinically relevant depressive symptoms at Year 2. Crude relative risk and its 95% CI for other potential confounding factors for this relationship were also calculated. These potential confounders included socio-demographic information (mean-centered age, education, marital status), lifestyle information (cigarette smoking, alcohol consumption and physical activity), medical conditions (cardiovascular disease, COPD, diabetes mellitus, stroke, BMI) and stressful life events that have been shown to be associated with depressive symptoms in the literature. Variables found to be associated with clinically relevant depressive symptoms in the initial uni-variate analyses were put into a multiple logistic regression model. Relative risk (95% CIs) for demographic, lifestyle, medical factors and the presence of moderate to severe LUTS in relation to having clinically relevant depressive symptoms were calculated by multiple logistic regression.

All statistical analyses were performed using the statistical package SAS, version 8.02 (SAS Institute, Inc., Cary, North Carolina).

## Results

### Baseline Characteristics

**Figure 1:**
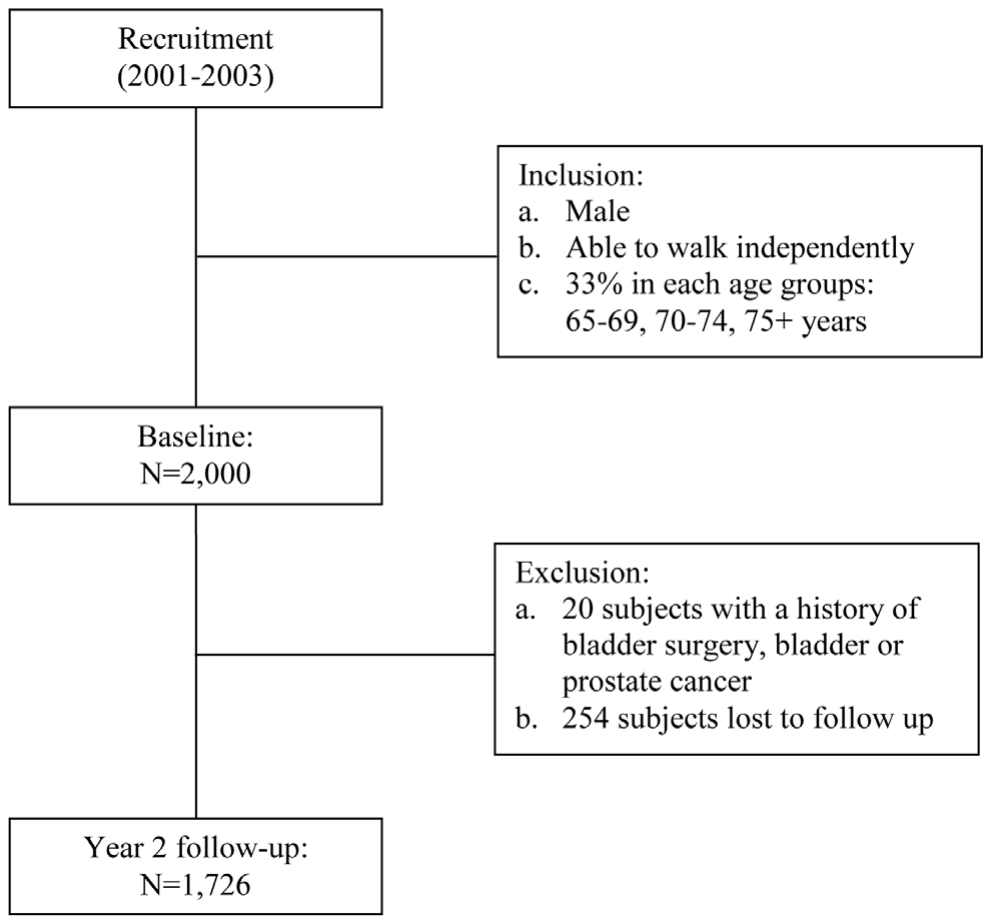
Flow of the study subjects.

The results of the baseline demographics are presented in Table 1. Of the 2,000 men recruited, 20 were excluded as they had either a history of bladder surgery or had a history of bladder or prostate cancer. 254 subjects were lost to follow-up at Year 2 with a retention rate of 87%. After exclusion, 1,726 subjects remained for analyses (Figure 1).

**Table 1:** Subjects’ characteristics at baseline (n = 1,726).

Variable	Mean (SD)/N (%)
Age (year)	72.1 (4.8)
Geriatric Depression Scale (score)	2.8 (2.7)
Geriatric Depression Scale (score) at year 2 follow-up	1.8 (2.4)
Lower urinary tract symptoms	
None (0)	90 (5.2%)
Mild (1–7)	950 (55.0%)
Moderate (8–19)	565 (32.7%)
Severe (20–35)	121 (7.0%)
Marital status	
Married of living in a married-like relationship	1,528 (88.5%)
Widowed	131 (7.6%)
Separated	19 (1.1%)
Divorced	14 (0.8%)
Single, never married	34 (2.0%)
Education	
Primary or below	1,026 (59.4%)
Secondary/Matriculation	457 (26.5%)
University or above	243 (14.1%)
PASE	
<60.4	399 (23.1%)
60.4–<89.3	439 (25.4%)
89.3–<124.6	435 (25.2%)
124.6 or above	453 (26.3%)
CHD	267 (15.5%)
COPD	187 (10.8%)
Diabetes	249 (14.4%)
Current smoker	197 (11.4%)
Drink >12 alcoholic drinks in past 12 m	415 (24.0%)
Beta-blocker	270 (15.6%)
Anti-depressants	8 (0.5%)
BMI	
Normal (18.5–<23)	654 (37.9%)
Overweight or Obese (23 or above)	985 (57.1%)
Underweight (<18.5)	87 (5.0%)
Number of stressful life event >1 at year 2 follow-up	393 (22.8%)

At baseline, the majority (88.5%) of men were married or living with a partner. More than half (59.4%) had only a primary or lower education. Overall, 11.4% were current smokers with about half (52.3%) having a past history of smoking. Also, most subjects (76.0%) were non-drinkers.

The overall mean (and its standard deviation) GDS score was 2.8 (2.7) at baseline, and 1.8 (2.4) at Year 2. Moreover, the mean (and its standard deviation) GDS score for the controls and the depressed cases was 2.7 (2.6) and 6.8 (3.4), respectively at baseline, and 1.5 (1.8) and 9.6 (1.7), respectively at Year 2.

### LUTS and Clinically Relevant Depressive Symptoms

The associations between lifestyle, medical and socio-demographic factors and the presence of clinically relevant depressive symptoms are shown in Table 2. In the univariate analysis, the presence of moderate to severe LUTS at baseline was associated with increased risk of having clinically relevant depressive symptoms at Year 2 (OR = 3.25; CI: 1.91–5.52). Also, having a history of coronary heart disease, COPD and diabetes were associated with increased risk of having clinically relevant depressive symptoms (OR = 2.04; CI: 1.15–3.62 for having a history of coronary heart disease; OR = 1.96; CI: 1.03–3.74 for having a history of COPD; and OR = 1.87; CI: 1.03–3.39 for having diabetes). In the multivariable analysis after adjusting for other co-variables and baseline clinically relevant depressive symptoms, having moderate to severe LUTS at baseline (OR = 2.50; CI: 1.41–4.42) still remained to be significantly associated with increased risk of having clinically relevant depressive symptoms at Year 2. Being depressed at baseline (OR = 5.61; CI: 3.07–10.25), having diabetes (OR = 2.01; CI: 1.04–3.88), and having more than one stressful life events (OR = 2.43;CI: 1.41–4.20) were also independently associated with increased risk of having clinically relevant depressive symptoms at Year 2.

**Table 2:** Crude odds ratio (univariate logistic regression) and adjusted odds ratio (multiple logistic regression) for having clinically relevant depressive symptoms at Year 2 follow-up in relation to demographic, lifestyle, medical factors, weight status, stressful life events and baseline depressive symptoms.

Variable	Percentage (%)		Crude Odds ratio(95% CI)	Adj. Odds ratio(95% CI)
	Controls(N = 1662)	Depressed(N = 64)		
Lower urinary tract symptoms				
None-Mild (0–7)	61.3	32.8	1	1
Moderate-Severe (8–35)	38.7	67.2	**3.25 (1.91, 5.52)**	**2.50 (1.41, 4.42)**
Age (year)	72.1 (4.8)	72.6(4.8)	1.02 (0.97, 1.07)	0.99 (0.94, 1.05)
Marital status				
Married of living in a married-like relationship	88.7	84.4	1	1
Widowed	7.6	7.8	1.08 (0.43, 2.76)	0.96 (0.34, 2.66)
Separated	1.0	3.1	3.21 (0.72, 14.25)	4.00 (0.83, 19.21)
Divorced	0.8	1.6	2.10 (0.27, 16.34)	1.17 (0.11, 12.45)
Single, never married	1.9	3.1	1.71 (0.40, 7.30)	0.83 (0.17, 4.15)
Education				
Primary or below	59.2	65.6	1	1
Secondary/Matriculation	26.6	23.4	0.80 (0.44, 1.45)	0.90 (0.48, 1.70)
University or above	14.2	10.9	0.70 (0.31, 1.57)	0.87 (0.37, 2.09)
PASE				
<60.4	22.4	40.6	1	1
60.4–<89.3	25.9	12.5	**0.27 (0.12, 0.60)**	**0.31 (0.14, 0.72)**
89.3–<124.6	25.3	21.9	**0.48 (0.25, 0.93)**	0.53 (0.26, 1.08)
124.6 or above	26.3	25.0	**0.53 (0.28, 0.99)**	0.60 (0.30, 1.19)
CHD	15.0	26.6	**2.04 (1.15, 3.62)**	1.37 (0.72, 2.60)
COPD	10.5	18.8	**1.96 (1.03, 3.74)**	1.66 (0.82, 3.36)
Diabetes	14.1	23.4	**1.87 (1.03, 3.39)**	**2.01 (1.04, 3.88)**
Depressed at baseline	6.4	34.4	**7.61 (4.38, 13.22)**	**5.61 (3.07, 10.25)**
Current smoker	11.5	9.4	0.80 (0.34, 1.87)	0.62 (0.25, 1.58)
Drink >12 alcoholic drinks in past 12 m	24.3	18.8	0.72 (0.38, 1.36)	0.88 (0.44, 1.74)
Beta-blocker	15.4	21.9	1.54 (0.84, 2.82)	1.20 (0.59, 2.42)
Anti-depressants	0.4	1.6	3.75 (0.45, 30.96)	1.65 (0.16, 17.44)
BMI				
Normal (18.5–<23)	37.7	42.2	1	1
Overweight or Obese (23 or above)	57.3	51.6	0.81 (0.48, 1.35)	0.67 (0.38, 1.20)
Underweight (<18.5)	5.0	6.3	1.12 (0.38, 3.28)	0.93 (0.28, 3.03)
Number of stressful life event >1 at year 2 follow-up	22.1	40.6	**2.41 (1.45, 4.03)**	**2.43 (1.41, 4.20)**
				R^2^ = 0.1771

## Discussion

LUTS have been shown to significantly affect the daily functioning of men; however, to our knowledge, this is the first prospective study in Asia that has demonstrated a temporal association between the presence of moderate-to-severe LUTS at baseline and increased risk of clinically relevant depressive symptoms after a period of 2 years, adjusting for other confounders including demographic, lifestyle, medical factors, weight status, stressful life events as well as baseline depressive symptoms.

A recent Japanese study investigated the link between LUTS and depression, but found no significant association between the two in contrast to the present study [21]. This is probably due to small sample size and selection bias of that study, because recruitments were only done on patients with late-onset hypogonadism, of which LUTS is not a typical symptom according to the guidelines set forth by the International Society for the Study of the Aging Male. Nevertheless, some other non-Asian studies have shown similar results. In a recent large nested case-control study, Coyne and colleagues [22] showed that overactive bladder, incontinence and other LUTS were associated with multidimensional impact on patients that included decreased quality of life, greater symptom bother, decreased enjoyment of sexual activity and higher rates of depression. In another study, Fitzgerald and colleagues [23] showed that all urologic symptoms were significantly related to at least one other medical condition, with the presence of depression associated with increased odds of all urologic and sexual symptoms. On the other hand, Laumann and colleagues [24] showed that the association of LUTS and depressive symptoms in men may also be bi-directional. While the present study has shown LUTS as a risk factor for depressive symptoms, our previous prospective study [25] has also demonstrated that depressive symptoms may increase the risk of LUTS in Chinese elderly men. Therefore, the association between LUTS and depressive symptoms may very well be reciprocal.

There are several mechanisms in which LUTS can lead to manifesting depressive symptoms in Chinese elderly men, mainly due to demoralization of having LUTS. First, in terms of functional impact of having LUTS, LUTS can lead to depression through the feeling of bother, because the patients are discouraged of performing normal daily activities [10], and great impairment in daily activities is a strong risk factor of major depression [26]–[27]. For men being married or living in a married-like relationship, sexual dysfunction [28]–[30] can be another possible mechanism linking LUTS to depression. Second, in terms of the psychological impact of having LUTS, men with LUTS tend to be embarrassed and socially anxious about their conditions [12], and some urological patients may hold negative views toward having prostatic diseases [31]. Third, LUTS may lead to depression indirectly through sleep deprivation, as nocturia, one of the major LUTS, is associated with excessive daytime sleepiness, decreased ability to perform tasks which need attention, as well as depression [32].

While we have added to the literature that the presence of LUTS could lead to increased risk of having clinically relevant depressive symptoms, whether treating the LUTS could result in better mood and decreased in the risk of having depressive symptoms is unknown. In a recent study, Quek and colleagues [33] have shown that medical and surgical treatments that improve patients’ LUTS could also improve their overall mood symptoms including anxiety and depression. However, that study was not a randomized controlled trial and only pre- and post- intervention design was employed. Nevertheless, since the relationship between LUTS and depression may be bi-directional, as suggested together by our present and previous study [24], any intervention that may improve the LUTS situation may be beneficial to the mood of the patients, which may then feed back to improve the LUTS situation through possible suggested pathways, including decreased adrenergic tone [24], common neurotransmitter responsible for both urological and depressive symptoms [23], [34], as well as the phosphodiesterase isozymes [24], [35]. Further intervention studies need to be conducted to confirm this hypothesis, which can have important public health impact to the elderly suffering from both urological and psychological problems.

Our study has several limitations. First, only a screening instrument, the GDS, was used to facilitate assessment of clinically relevant depressive symptoms in older adults instead of a physician-administered psychiatric diagnostic interview. While the prevalence of depression could have been over-estimated due to the instrument’s high sensitivity and specificity [36], the GDS is still useful as a quick and easy instrument to screen for depression among older adults. Second, the study sample consisted of all volunteers; as a result, it is possible that our sample is healthier than that recruited from the general population because the recruitment of subjects from community centers would generally consist of a healthier group of older adults, be it psychologically or physically when compared to that of the general population. People with severe LUTS may also find it inconvenient to take part in this study, since it requires the subjects to travel to the study site. Third, only 2.6% of the subjects developed depressive symptoms over the 2-year follow-up period. While we are cautious with our interpretations of the results, 2.6% of all people suffering from LUTS in the population is not a small number; thus, we cannot undermine the public health relevance of our findings in a population level. Moreover, our sample has shown to be generally more educated with greater awareness of health [37]. Therefore, we are cautious with our interpretations of the results. Last, we used interviewers to conduct the IPSS questionnaires on the subjects, who may find embarrassing to disclose information on their LUTS in front of other people. However, this may be the only feasible method of administration because many older adults in Hong Kong have only attained education of the primary level or below (∼60% of subjects in this study). In addition, only two subjects refused to answer questions related to LUTS; hence, it is unlikely that our results were made biased in this way.

## Conclusion

In conclusion, depression is more common in patients with moderate to severe LUTS when compared to those without. As elderly are at increased risk of depression, mental illness and suicide, men who suffer from moderate to severe LUTS may be at even higher risk. Clinicians and health care workers in both primary and urologic settings should be alert to the increased prevalence of clinically depressive symptoms in this high risk population. Due to the possible bi-directionality of the relationship between LUTS and depression, any intervention that improves the LUTS situation may also be beneficial towards reducing depressive symptoms, which may then feed back to improve the LUTS situation. The findings warrant further investigations.

## References

[1] Whooley MA (2006) Depression and cardiovascular disease. JAMA 295: 2874–2881. 16804154 10.1001/jama.295.24.2874 PMC2771193

[2] Ng TP, Niti M, Tan WC, Cao Z, Ong KC, et al (2007) Depressive symptoms and chronic obstructive pulmonary disease: effect on mortality, hospital readmission, symptom burden, functional status, and quality of life. Arch Intern Med 167: 60–67. 17210879 10.1001/archinte.167.1.60

[3] Sherwood A, Blumenthal JA, Trivedi R, Johnson KS, O’Connor CM, et al (2007) Relationship of depression to death or hospitalization in patients with heart failure. Arch Intern Med 167: 367–373. 17325298 10.1001/archinte.167.4.367

[4] Sundquist J, Li X, Johansson SE, Sundquist K (2005) Depression as a predictor of hospitalization due to coronary heart disease. Am J Prev Med 29: 428–433. 16376706 10.1016/j.amepre.2005.08.002

[5] Bula CJ, Wietlisbach V, Burnand B, Yersin B (2001) Depressive symptoms as a predictor of a 6 month outcomes and services utilization in elderly medical inpatients. Arch Intern Med 161: 2609015. 10.1001/archinte.161.21.2609 11718593

[6] Kirby RS (2000) The natural history of benign prostatic hyperplasia: What have we learned in the last decade? Urology 56: 3–6. 11074195 10.1016/s0090-4295(00)00747-0

[7] Platz EA, Smit E, Curhan GC, Nyberg LM, Giovannucci E (2002) Prevalence of and racial/ethnic variation in lower urinary tract symptoms and noncancer prostate surgery in U.S. men. Urology 59: 877–883. 12031373 10.1016/s0090-4295(01)01673-9

[8] Trueman P, Hood SC, Nayak US, Mrazek MF (1999) Prevalence of lower urinary tract symptoms and self-reported diagnosed “benign prostatic hyperplasia” and their effect on quality of life in a community-based survey of men in the UK. BJU Int 83: 410–415. 10210562 10.1046/j.1464-410x.1999.00966.x

[9] Bertaccini A, Vassallo F, Martino F, Luzzi L, Rocca Rossetti S, et al (2001) Symptoms, bothersomeness and quality of life in patients with LUTS suggestive of BPH. Eur Urol 40: 13–18. 10.1159/000049872 11598348

[10] Eckhardt MD, van Venrooij GE, van Melick HH, Boon TA (2001) Prevalence and bothersomeness of lower urinary tract symptoms in benign prostatic hyperplasia and their impact on well-being. J Urol 166: 563–568. 11458069

[11] Welch G, Weinger K, Barry MJ (2002) Quality of life impact of lower urinary tract symptom severity: results from the Health Professionals Follow-up Study. Urology 59: 245–250. 11834396 10.1016/s0090-4295(01)01506-0

[12] Glover L, Gannon K, McLoughlin J, Emberton M (2004) Men’s experiences of having lower urinary tract symptoms: factors relating to bother. BJU Int 94: 563–567. 15329113 10.1111/j.1464-410X.2004.05001.x

[13] Engstrom G, Henningsohns L, Stenieck G, Lepper J (2005) Self-assessed health, sadness and happiness in relation to the total burden of symptoms from the lower urinary tract. BJU Int 95: 810–815. 15794788 10.1111/j.1464-410X.2005.05406.x

[14] Irwin DE, Milsom I, Kopp Z, Abrams P, Cardozo L (2006) Impact of overactive bladder symptoms on employment, social interactions and emotional well being in six European countries. BJU Int 97: 96–100. 16336336 10.1111/j.1464-410X.2005.05889.x

[15] Wong SY, Hong A, Leung J, Kwok T, Leung PC, et al (2006) Lower urinary tract symptoms and depressive symptoms in elderly men. J Affect Disord 96: 83–88. 16815555 10.1016/j.jad.2006.05.013

[16] Wong SY, Kwok T, Woo J, Lynn H, Griffith JF, et al (2005) Bone Mineral Density and the Risk of Peripheral Arterial Disease in men and women: Results from Mr and Ms Os, Hong Kong. Osteoporos Int 16: 1933–1938. 16079958 10.1007/s00198-005-1968-3

[17] Chan HC (2004) The psychometric evaluation of the Chinese version of the international prostate symptom score (IPSS). [Thesis] Hong Kong: Hong Kong University.

[18] Lee HB, Chiu HFK, Kwok WY, Leung CM, Kwong PK, et al (1993) Chinese elderly and the GDS short form: a preliminary study. Clin Geront 14: 37–39.

[19] Lau EM, Chan HH, Woo J, Lin F, Black D, et al (1996) Normal ranges for vertebral height ratios and prevalence of vertebral fracture in Hong Kong Chinese: A comparison with American Caucasians. J Bone Miner Res 11: 1364–1368. 8864912 10.1002/jbmr.5650110922

[20] Washburn RA (1993) The physical activity scale for the elderly (PASE): Development and Evaluation. J Clin Epidemiol 46: 153–162. 8437031 10.1016/0895-4356(93)90053-4

[21] Takao T, Tsujimura A, Okuda H, Yamamoto K, Fukuhara S, et al. Lower urinary tract symptoms and erectile dysfunction associated with depression among Japanese patients with late-onset hypogonadism symptoms. Aging Male 14: 110–114. 20828247 10.3109/13685538.2010.512374

[22] Coyne KS, Sexton CC, Irwin DE, Kopp ZS, Kelleher CJ, et al (2008) The impact of overactive bladder, incontinence and other lower urinary tract symptoms on quality of life, work productivity, sexuality and emotional well being in men and women: results from the EPIC study. BJU Int 101: 1388–1395. 18454794 10.1111/j.1464-410X.2008.07601.x

[23] Fitzgerald MP, Link CL, Litman HJ, Travison TG, McKinlay JB (2007) Beyond the lower urinary tract: the association of urologic and sexual symptoms with common illnesses. Eur Urol 52: 407–415. 17382458 10.1016/j.eururo.2007.03.014 PMC2396456

[24] Laumann EO, Kang JH, Glasser DB, Rosen RC, Carson CC (2008) Lower urinary tract symptoms are associated with depressive symptoms in White, Black and Hispanic men in the United States. J Urol 180: 233–240. 18499181 10.1016/j.juro.2008.03.055

[25] Wong SY, Woo J, Leung JC, Leung PC (2010) Depressive symptoms and lifestyle factors as risk factors of lower urinary tract symptoms in Southern Chinese men: a prospective study. Aging Male 13: 113–119. 20429720 10.3109/13685530903440432

[26] Chi I, Yip PS, Chiu HF, Chou KL, Chan KS, et al (2005) Prevalence of depression and its correlates in Hong Kong’s Chinese older adults. Am J Geriatr Psychiatry 13: 409–416. 15879590 10.1176/appi.ajgp.13.5.409

[27] Chou KL, Chi I (2005) Prevalence and correlates of depression in Chinese oldest-old. Int J Geriatr Psychiatry 20: 41–50. 15578666 10.1002/gps.1246

[28] Wong SY, Chan D, Hong A, Leung PC, Woo J (2006) Depression and lower urinary tract symptoms: Two important correlates of erectile dysfunction in middle-aged men in Hong Kong, China. Int J Urol 13: 1304–10. 17010009 10.1111/j.1442-2042.2006.01560.x

[29] Rosen RC (2004) Lower urinary tract symptoms and sexual dysfunction: additional evidence of an association. BJU Int 93: 689–94. 15049970 10.1111/j.1464-410X.2003.04767.x

[30] Araujo AB, Johannes CB, Feldman HA, Derby CA, McKinlay JB (2000) Relation between psychosocial risk factors and incidence of erectile dysfunction: prospective results from the Massachusetts male aging study. Am J Epidemiol 152(6): 533–41. 10997543 10.1093/aje/152.6.533

[31] Gannon K, Glover L, O’Neill M, Emberton M (2005) Lower urinary tract symptoms in men: self perceptions and the concept of bother. BJU Int 96: 823–827. 16153210 10.1111/j.1464-410X.2005.05720.x

[32] Marschall-Kehrel D (2004) Update on nocturia: the best of rest is sleep. Urology 64: 21–24. 15621224 10.1016/j.urology.2004.10.072

[33] Quek KF, Razack AH, Chua CB, Low WY, Loh CS (2004) Effect of treating lower urinary tract symptoms on anxiety, depression and psychiatric morbidity: a one-year study. Int J Urol 11: 848–855. 15479289 10.1111/j.1442-2042.2004.00903.x

[34] Mariappan P, Ballantyne Z, N’Dow JM, Alhasso AA (2005) Serotonin and noradrenaline reuptake inhibitors (SNRI) for stress urinary incontinence in adults. Cochrane Database Syst Rev (3): CD004742. 10.1002/14651858.CD004742.pub2 PMC12699447 16034945

[35] Roehrborn CG (2004) Lower urinary tract symptoms, benign prostatic hyperplasia, erectile dysfunction, and phosphodiesterase-5 inhibitors. Rev Urol 6: 121–127. 16985592 PMC1472825

[36] Marc LG, Raue PJ, Bruce ML. Screening Performance of the Geriatric Depression Scale (GDS-15) in a Diverse Elderly Home Care Population (2008) Am J Geriatr Psychiatry. 16: 914–921. 10.1097/JGP.0b013e318186bd67 PMC2676444 18978252

[37] Wong SY, Lau WW, Leung PC, Leung JC, Woo J (2007) The association between isoflavone and lower urinary tract symptoms in elderly men. Br J Nutr 98: 1237–1242. 17640419 10.1017/S0007114507787433

